# Nomogram combining clinical and ultrasonographic features for differentiating granulomatous lobular mastitis from invasive lobular carcinoma

**DOI:** 10.3389/fonc.2026.1915144

**Published:** 2026-08-20

**Authors:** Yuqing Zhang, Mei Wu, Ya Li, Yan Li

**Affiliations:** 1 Department of Ultrasound, The Second Qilu Hospital of Shandong University, Jinan, Shandong, China; 2 Department of Nuclear Medicine, The Second Qilu Hospital of Shandong University, Jinan, Shandong, China

**Keywords:** breast, clinical features, granulomatous lobular mastitis, invasive lobular carcinoma, nomogram model, ultrasonographic features

## Abstract

**Objective:**

This study aims to develop and validate a nomogram model based on clinical and ultrasonographic features for differentiating granulomatous lobular mastitis (GLM) from invasive lobular carcinoma (ILC) of the breast in clinical practice and evaluate its diagnostic performance and clinical utility.

**Methods:**

A total of 205 patients with pathologically confirmed GLM and 166 patients with ILC were retrospectively enrolled and randomly allocated to training and validation cohorts in a 7:3 ratio. Clinical data and ultrasonographic characteristics were collected. Univariate analysis was performed to compare variables between GLM and ILC groups. Variables with statistical significance were subsequently entered into multivariate logistic regression analysis to identify independent predictors. A visual nomogram model was constructed based on these predictors. Model performance was evaluated using receiver operating characteristic curves and the area under the curve. Calibration curves were plotted to evaluate the agreement between predicted probabilities and observed outcomes. Decision curve analysis (DCA) was conducted to assess the clinical net benefit of the model.

**Results:**

Univariate analysis revealed significant differences across several clinical and ultrasonographic features between GLM and ILC (*P* < 0.05). Within the training cohort, multivariate logistic regression identified age category, menopausal status, lesion classification, shape, margin characteristics, posterior echo, and axillary lymph nodes enlarged as independent predictors for distinguishing GLM from ILC. The nomogram derived from these variables exhibited strong predictive capability, with AUCs of 0.97 and 0.96 for the training and validation cohorts, respectively. Calibration curves exhibited good agreement between predicted probabilities and observed outcomes. Furthermore, DCA demonstrated a substantial net clinical benefit associated with the model.

**Conclusion:**

A nomogram integrating clinical and ultrasonographic features was developed and validated, demonstrating robust discrimination, satisfactory calibration, and favorable clinical utility in differentiating GLM from ILC. As a noninvasive and visually intuitive tool, this model requires external validation, it still may facilitate more precise diagnostic and therapeutic decision-making.

## 1 Introduction

Granulomatous lobular mastitis (GLM) is a chronic inflammatory disorder of unknown etiology characterized by non-caseating granulomatous inflammation predominantly involving the breast lobules (1, 2). Initially described by Kessler and Wolloch in 1972, the diagnosis of GLM requires the exclusion of identifiable causes of granulomatous lesions, including tuberculosis, fungal infections, sarcoidosis, and Wegener’s granulomatosis (3). GLM accounts for approximately 1.8% of benign breast diseases, and its incidence has been steadily increasing in recent years (4). It predominantly affects women of childbearing age, with a mean age ranging from 32 to 34 years across various studies (5).Invasive lobular carcinoma (ILC) represents the second most prevalent subtype of invasive breast cancer, accounting for 10%–15% of cases. A key pathological hallmark is the loss of E-cadherin-mediated cell adhesion, leading to diffuse infiltration of cancer cells arranged in single-file linear cords or small clusters (6).

Ultrasound examination, due to its real-time imaging capability, non-invasive characteristics, and high sensitivity in dense breast tissue, is widely utilized as a primary imaging modality for evaluating breast lesions (7, 8).Despite clear pathological differences between GLM and ILC, with GLM being a benign inflammatory condition that may even resolve spontaneously in some patients, their clinical manifestations are highly similar. Both conditions primarily occur in middle-aged women and commonly present as firm, ill-defined, poorly mobile masses, often accompanied by axillary lymph nodes enlarged (ALNE). This overlap in clinical phenotype poses significant diagnostic challenges and increases the risk of misdiagnosis (2). Previous studies have demonstrated that the sonographic features of GLM are heterogeneous and variable, including irregular, lobulated, or oval hypoechoic lesions with indistinct margins, as well as tubular or irregular anechoic areas and occasionally increased internal vascularity (9). Similarly, the sonographic features of ILC lack specificity, often presenting as hypoechoic masses with indistinct margins and, in some cases, increased internal vascularity. ILC lacks specific sonographic characteristics and often appears as hypoechoic lesions with indistinct margins and posterior acoustic shadowing. In certain cases, tumor infiltration may lead to architectural distortion without the formation of a discrete mass (6). The substantial overlap in imaging findings between GLM and ILC makes accurate differentiation using conventional ultrasound particularly challenging.

This study aimed to retrospectively evaluate the clinical and ultrasonographic characteristics of patients with pathologically confirmed GLM and ILC. The study further aimed to compare differences between these groups, identify independent differentiating factors through multivariate logistic regression analysis, develop a visual nomogram model, and evaluate its discriminatory performance and clinical utility using receiver operating characteristic (ROC) curves, calibration curves, and decision curve analysis (DCA). The results of this study may improve diagnostic accuracy, provide an individualized risk assessment approach, support clinical decision-making, and ultimately improve patient outcomes.

## 2 Materials and methods

### 2.1 Patient cohort and study design

**Figure 1:**
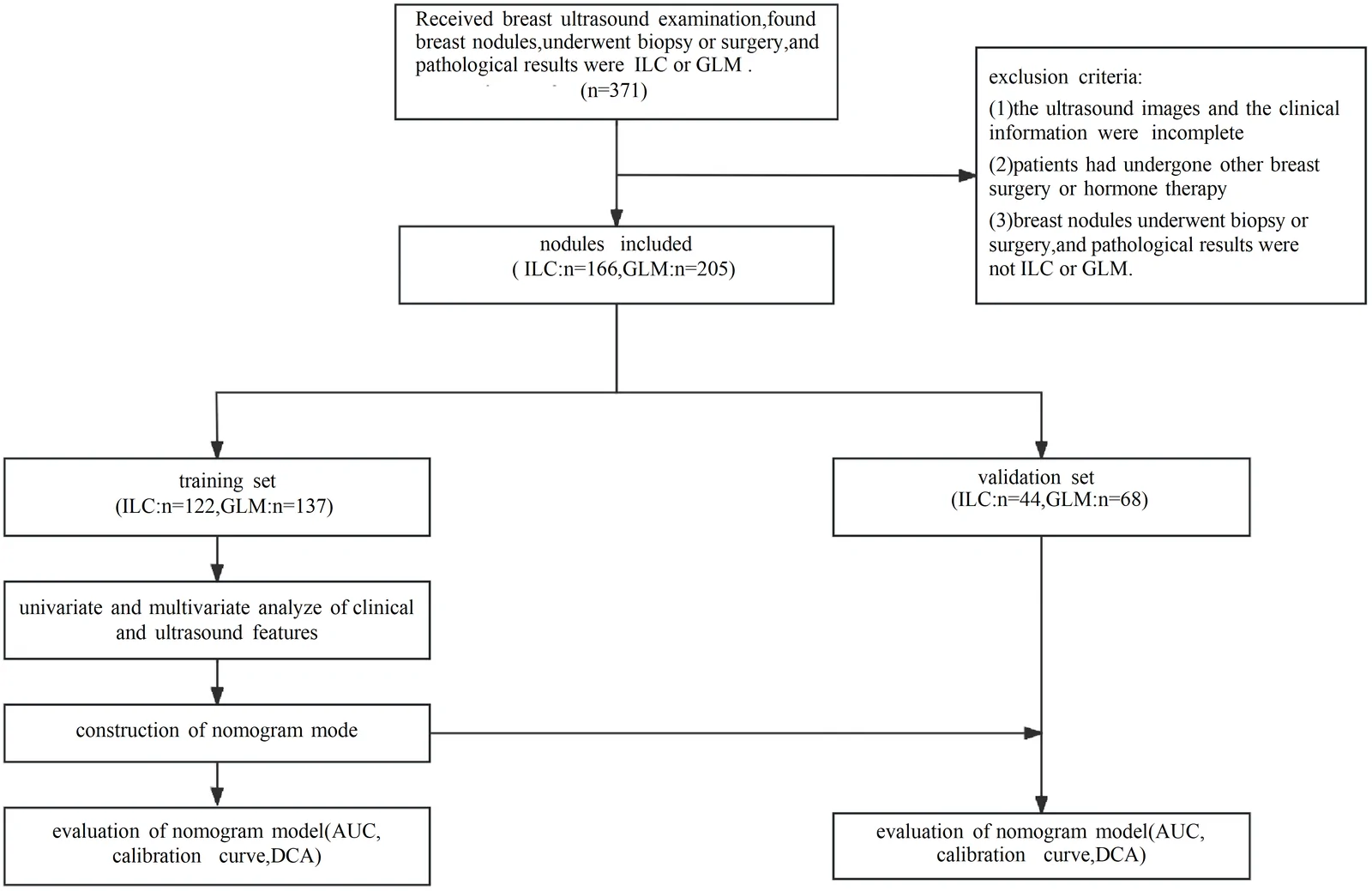
Flowchart of this study.

A retrospective analysis was conducted in patients diagnosed with GLM and ILC in the Department of Breast Surgery at the Second Qilu Hospital of Shandong University between January 2020 and December 2024. For patients with multifocal or multicentric disease, the largest lesion was designated as the index lesion for analysis. A total of 371 patients with 371 lesions were included. The inclusion criteria were as follows: (1) Complete clinical data and clear and high-quality ultrasound images; (2) histopathological confirmation of GLM or ILC obtained via surgical excision or biopsy specimens; (3) no history of prior radiotherapy, chemotherapy, or hormonal therapy. The exclusion criteria included: (1) Incomplete clinical data or suboptimal ultrasonographic image quality; (2) prior breast surgery or hormonal therapy before presentation; (3) histopathological diagnosis other than GLM or ILC. Clinical data were retrieved from the electronic medical record system. Ultrasonographic images were obtained from the Picture Archiving and Communication System (PACS). Patients were randomly allocated to a training cohort and a validation cohort in a 7:3 ratio. Cases with incomplete data for any key variable were excluded prior to analysis, resulting in a complete-case dataset with no missing values. This study was approved by the Institutional Review Board of the Second Qilu Hospital of Shandong University (Ethics Number: KYLL202604630). The participants provided their written informed consent to participate in this study. **Figure 1** illustrates the flowchart of the study.

### 2.2 Instruments and methods

Clinical information, including age, disease duration (interval between symptom onset and clinical presentation), menopausal status, clinical symptoms, lesion multiplicity, and palpability, was collected from the electronic medical record system.

Ultrasound examinations were performed using LOGIQ E9/Fortis (GE Healthcare, Chicago, IL, USA), Resona 7S, and Eagus R9S (Mindray, Shenzhen, China) diagnostic ultrasound systems equipped with high-frequency linear array transducers (9–18 MHz). Patients were examined in the supine position, and both transverse and longitudinal scans of the breast and axillary region were performed. Two-dimensional ultrasonographic features of each lesion were assessed and documented according to the Breast Imaging Reporting and Data System lexicon. The recorded parameters included lesion dimensions (length, width, and depth), lesion classification(non-mass lesions and mass lesions), ductalabnormality, echogenicity echotexture, internal echo, shape, hyperechoic halo, margin, posterior echo, calcification, aspect ratio, color Doppler flow imaging (CDFI), and axillary lymph nodes enlarged (ALNE). Vascularity was graded semi-quantitatively based on Adler classification (10): Grade 0: no flow; Grade I: minimal flow (1–2 punctate or short rod-like signals); Grade II: moderate flow (one long vessel or 3–4 punctate signals); Grade III: marked flow (two long vessels or ≥ 5 punctate signals). All ultrasonographic images were archived in the departmental PACS. Image interpretation was independently performed by two radiologists with more than 10 years of experience in breast imaging, both of whom were blinded to the pathological diagnoses. To evaluate interobserver reliability of ultrasound feature interpretation, 60 randomly selected images were independently reviewed by two senior sonographers (Expert A and B). Agreement was assessed using Cohen’s kappa, with kappa > 0.75 indicating good agreement. Discrepancies were resolved by consensus, with consultation from a senior physician when necessary.

### 2.3 Statistical analysis

Statistical analyses were conducted using R software (version 4.3.3). Continuous variables with normal distribution are presented as mean ± standard deviation (mean ± SD), whereas non-normally distributed variables are presented as median (interquartile range). Comparisons of continuous variables were performed using the independent-samples t-test for normally distributed data or the Mann–Whitney U test for non-normally distributed data. Categorical variables were compared using the Pearson χ^2^ test. Multivariate analysis was conducted using binary Logistic regression. *P* < 0.05 was considered statistically significant. To comprehensively evaluate the strength of association between each variable and the outcome, univariate binary logistic regression analysis was performed with pathological outcome (GLM or ILC) as the dependent variable. All clinical and ultrasound features were individually entered as independent variables, and the odds ratio (OR) and 95% confidence interval (CI) for each variable were calculated. In addition, baseline characteristics between the two groups were compared using independent-samples t-tests for continuous variables and χ² tests for categorical variables.ROC curves were generated, and the area under the curve (AUC) with 95% confidence intervals (CIs) was constructed to assess agreement between predictive performance and observed outcomes. Calibration curves were constructed to evaluate model calibration. DCA was performed to assess the clinical net benefit of the model. The sample size estimation met the criterion of events per variable (EPV) ≥ 10. With 122 ILC events in the training set and seven predictors included in the final model, the EPV was 17.4. Model robustness was evaluated by internal validation using bootstrap resampling (1,000 resamples).

## 3 Results

### 3.1 Comparison of clinical and ultrasonographic features between training and validation sets

A total of 371 patients were included and randomly allocated to training (n = 259) and validation (n = 112) cohorts. No statistically significant differences were identified in clinical and ultrasonographic characteristics between the two sets (*P* > 0.05), indicating comparability between groups. Detailed baseline characteristics are presented in **Table 1**.

**Table 1:** Baseline table.

Variables	Total (n = 371)	Train (n = 259)	Test (n = 112)	T/χ^2^	*P*
Age (years)	41.92 ± 11.82	41.88 ± 11.66	42.02 ± 12.25	0.10	0.921
Length (cm)	1.68 ± 3.59	1.62 ± 3.48	1.81 ± 3.87	0.46	0.643
Width (cm)	3.51 ± 2.44	3.44 ± 2.46	3.66 ± 2.40	0.78	0.437
Depth (cm)	2.51 ± 1.82	2.43 ± 1.77	2.68 ± 1.93	t1.21	0.226
Volume (cm^3^)	1.48 ± 0.93	1.46 ± 0.93	1.53 ± 0.92	0.60	0.550
L/D	28.89 ± 66.33	27.11 ± 61.02	33.00 ± 77.38	0.78	0.433
Age groups, n (%)				0.22	0.636
≤ 35 years	109 (29.38)	78 (30.12)	31 (27.68)		
> 35 years	262 (70.62)	181 (69.88)	81 (72.32)		
Menopause, n (%)				0.07	0.798
Yes	288 (77.63)	202 (77.99)	86 (76.79)		
No	83 (22.37)	57 (22.01)	26 (23.21)		
Clinical symptoms, n (%)				0.49	0.484
No	43 (11.59)	32 (12.36)	11 (9.82)		
Yes	328 (88.41)	227 (87.64)	101 (90.18)		
Multiple lesions, n (%)				0.23	0.630
No	268 (72.24)	189 (72.97)	79 (70.54)		
Yes	103 (27.76)	70 (27.03)	33 (29.46)		
Palpable nodule, n (%)				0.56	0.453
No	82 (22.10)	60 (23.17)	22 (19.64)		
Yes	289 (77.90)	199 (76.83)	90 (80.36)		
Lesion classification, n (%)				0.52	0.471
NML	109 (29.38)	79 (30.50)	30 (26.79)		
ML	262 (70.62)	180 (69.50)	82 (73.21)		
Ductal abnormality, n (%)				1.18	0.278
No	324 (87.33)	223 (86.10)	101 (90.18)		
Yes	47 (12.67)	36 (13.90)	11 (9.82)		
Aspect ratio, n (%)				0.31	0.579
< 1	340 (91.64)	236 (91.12)	104 (92.86)		
≥ 1	31 (8.36)	23 (8.88)	8 (7.14)		
Echotexture, n (%)				3.30	0.192
Hyperechoic	18 (4.85)	15 (5.79)	3 (2.68)		
Hypoechoic	268 (72.24)	190 (73.36)	78 (69.64)		
Mixed	85 (22.91)	54 (20.85)	31 (27.68)		
Internal echo, n (%)				0.08	0.780
Heterogeneity	6 (1.62)	5 (1.93)	1 (0.89)		
Homogeneity	365 (98.38)	254 (98.07)	111 (99.11)		
Shape, n (%)				0.62	0.431
Regular	51 (13.75)	38 (14.67)	13 (11.61)		
Irregular	320 (86.25)	221 (85.33)	99 (88.39)		
Margin, n (%)				0.01	0.953
Distinct	92 (24.80)	64 (24.71)	28 (25.00)		
Indistinct	279 (75.20)	195 (75.29)	84 (75.00)		
Posterior echo, n (%)				0.59	0.774
No features	153 (41.24)	105 (40.54)	48 (42.86)		
Enhancement	78 (21.02)	53 (20.46)	25 (22.32)		
Shadowing	140 (37.74)	101 (39.00)	39 (34.82)		
Echogenic halo, n (%)				0.11	0.743
No	301 (81.13)	209 (80.69)	92 (82.14)		
Yes	70 (18.87)	50 (19.31)	20 (17.86)		
Calcification, n (%)				2.66	0.264
Absent	269 (72.51)	183 (70.66)	86 (76.79)		
Microcalcifications	96 (25.88)	72 (27.80)	24 (21.43)		
Macrocalcification	6 (1.62)	4 (1.54)	2 (1.79)		
CDFI, n (%)				0.06	0.803
0-I	225 (60.65)	156 (60.23)	69 (61.61)		
II-III	146 (39.35)	103 (39.77)	43 (38.39)		
ALNE, n (%)				0.03	0.858
No	226 (60.92)	157 (60.62)	69 (61.61)		
Yes	145 (39.08)	102 (39.38)	43 (38.39)		
Pathology, n (%)				1.93	0.164
GLM	205 (55.26)	137 (52.90)	68 (60.71)		
ILC	166 (44.74)	122 (47.10)	44 (39.29)		

### 3.2 Analysis of clinical and ultrasonographic features in GLM and ILC groups within the training set

The training cohort comprised 137 patients with GLM and 122 patients with ILC. No statistically significant differences were found between the two groups regarding lesion length, internal echo homogeneity, hyperechoic halo, and CDFI grading (*P* > 0.05). Statistically significant differences were observed for all other clinical and ultrasonographic features (*P* < 0.05 for all). Detailed results are presented in **Table 2**.

**Table 2:** Univariate analysis of clinical and ultrasonographic features in the training set.

Variables	Total (n = 259)	GLM (n = 137)	ILC (n = 122)	T/χ^2^	*P*
Age (years)	41.88 ± 11.66	34.40 ± 6.61	50.29 ± 10.31	–14.56	**< 0.001**
Length (cm)	1.62 ± 3.48	1.59 ± 2.11	1.65 ± 4.56	–0.13	0.898
Width (cm)	3.44 ± 2.46	4.69 ± 2.55	2.05 ± 1.34	10.58	**< 0.001**
Depth (cm)	2.43 ± 1.77	3.18 ± 1.96	1.59 ± 1.00	8.40	**< 0.001**
Volume (cm^3^)	1.46 ± 0.93	1.73 ± 1.07	1.16 ± 0.63	5.34	**< 0.001**
L/D	27.11 ± 61.02	47.28 ± 77.99	4.47 ± 11.16	6.35	**< 0.001**
Age groups, n (%)				69.58	**< 0.001**
≤ 35 years	78 (30.12)	72 (52.55)	6 (4.92)		
> 35 years	181 (69.88)	65 (47.45)	116 (95.08)		
Menopause, n (%)				52.66	**< 0.001**
No	202 (77.99)	131 (95.62)	71 (58.20)		
Yes	57 (22.01)	6 (4.38)	51 (41.80)		
Clinical symptoms, n (%)				41.00	**< 0.001**
No	32 (12.36)	0 (0.00)	32 (26.23)		
Yes	227 (87.64)	137 (100.00)	90 (73.77)		
Multiple lesions, n (%)				9.46	**0.002**
No	189 (72.97)	89 (64.96)	100 (81.97)		
Yes	70 (27.03)	48 (35.04)	22 (18.03)		
Palpable nodule, n (%)				6.65	**0.001**
No	60 (23.17)	23 (16.79)	37 (30.33)		
Yes	199 (76.83)	114 (83.21)	85 (69.67)		
Lesion classification, n (%)				12.76	**< 0.001**
NML	79 (30.50)	55 (40.15)	24 (19.67)		
ML	180 (69.50)	82 (59.85)	98 (80.33)		
Ductal abnormality, n (%)				12.84	**< 0.001**
No	223 (86.10)	108 (78.83)	115 (94.26)		
Yes	36 (13.90)	29 (21.17)	7 (5.74)		
Aspect ratio, n (%)				28.34	**< 0.001**
< 1	236 (91.12)	137 (100.00)	99 (81.15)		
≥1	23 (8.88)	0 (0.00)	23 (18.85)		
Echotexture, n (%)				49.38	**< 0.001**
Hyperechoic	15 (5.79)	9 (6.57)	6 (4.92)		
Hypoechoic	190 (73.36)	77 (56.20)	113 (92.62)		
Mixed	54 (20.85)	51 (37.23)	3 (2.46)		
Internal echo, n (%)				3.77	0.052
Heterogeneity	5 (1.93)	0 (0.00)	5 (4.10)		
Homogeneity	254 (98.07)	137 (100.00)	117 (95.90)		
Shape, n (%)				10.25	0.001
Regular	38 (14.67)	11 (8.03)	27 (22.13)		
Irregular	221 (85.33)	126 (91.97)	95 (77.87)		
Margin, n (%)				52.67	**< 0.001**
Distinct	64 (24.71)	59 (43.07)	5 (4.10)		
Indistinct	195 (75.29)	78 (56.93)	117 (95.90)		
Posterior echo, n (%)				51.23	**< 0.001**
No features	105 (40.54)	66 (48.18)	39 (31.97)		
Enhancement	53 (20.46)	44 (32.12)	9 (7.38)		
Shadowing	101 (39.00)	27 (19.71)	74 (60.66)		
Echogenic halo, n (%)				0.65	0.421
No	209 (80.69)	108 (78.83)	101 (82.79)		
Yes	50 (19.31)	29 (21.17)	21 (17.21)		
Calcification, n (%)				39.86	**< 0.001**
Absent	183 (70.66)	120 (87.59)	63 (51.64)		
Microcalcifications	72 (27.80)	17 (12.41)	55 (45.08)		
Macrocalcification	4 (1.54)	0 (0.00)	4 (3.28)		
CDFI, n (%)				1.30	0.254
0-I	156 (60.23)	87 (63.50)	69 (56.56)		
II-III	103 (39.77)	50 (36.50)	53 (43.44)		
ALNE, n (%)				34.48	**< 0.001**
No	157 (60.62)	60 (43.80)	97 (79.51)		
Yes	102 (39.38)	77 (56.20)	25 (20.49)		

*Bold：P<0.05*

### 3.3 Multivariate logistic regression analysis and nomogram construction

**Figure 2:**
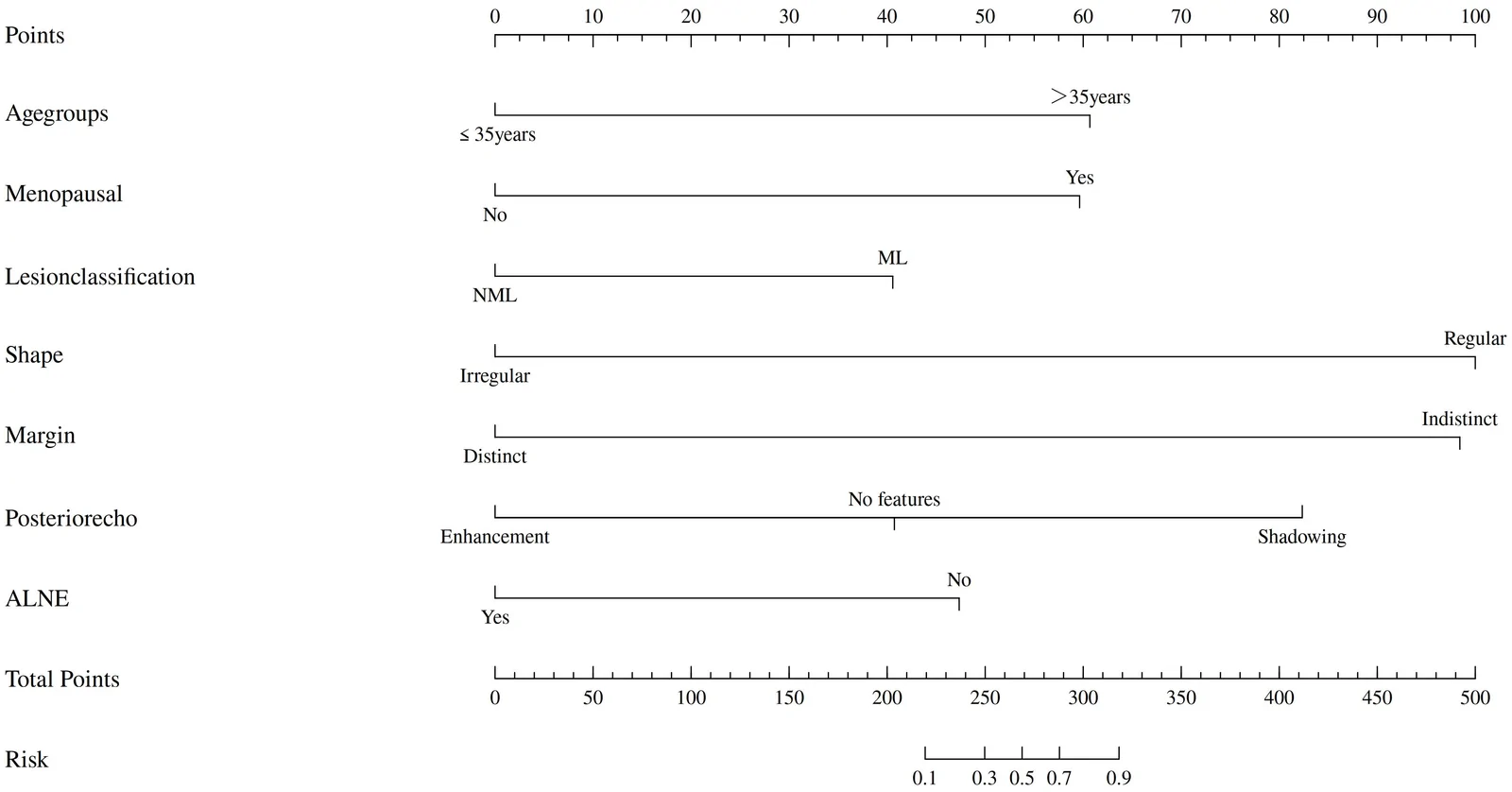
Nomogram for differentiating granulomatous lobular mastitis from invasive lobular carcinoma.

**Figure 3:**
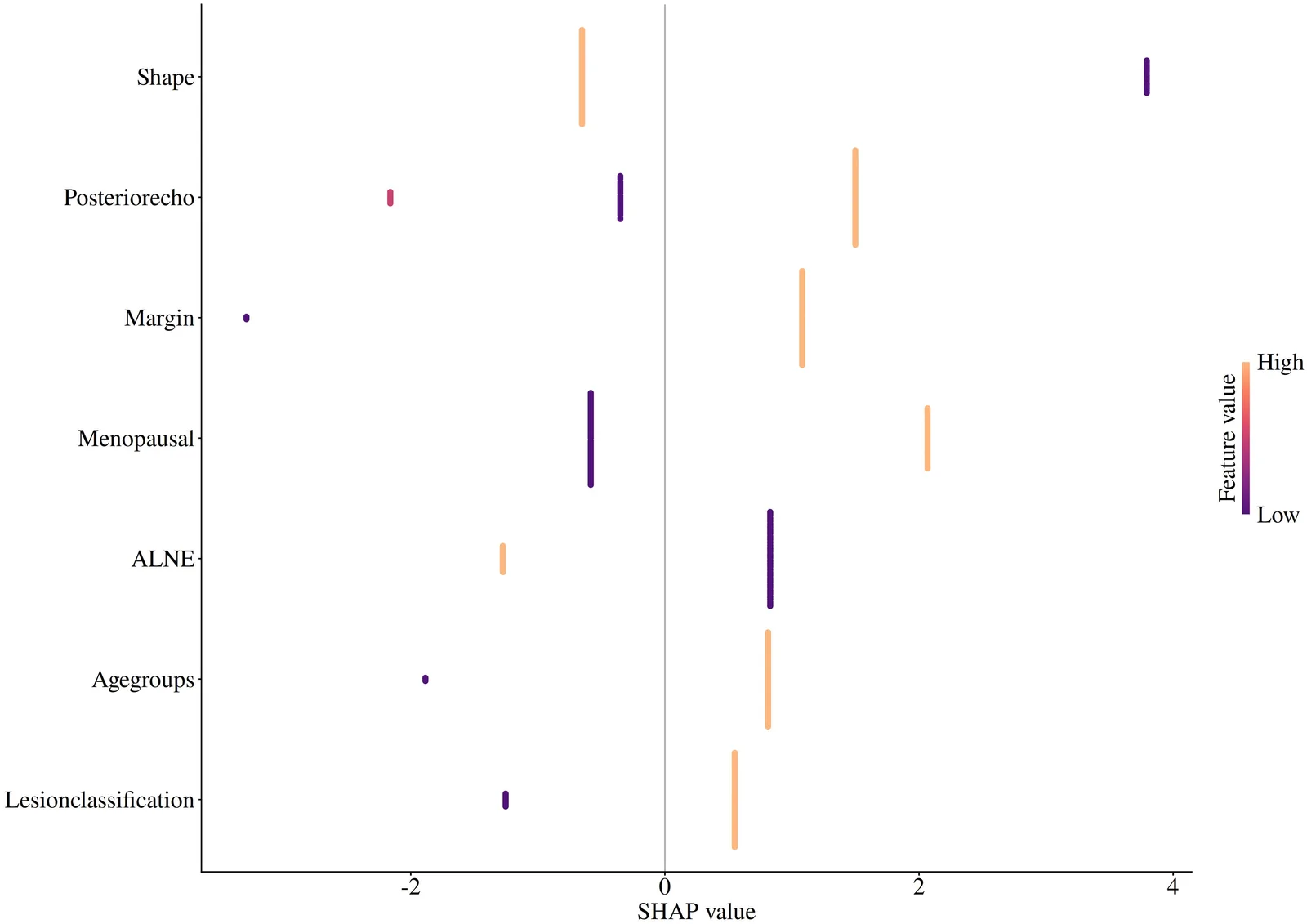
Model interpretability assessed using shapley additive explanations (SHAP): SHAP beeswarm plot.

**Figure 4:**
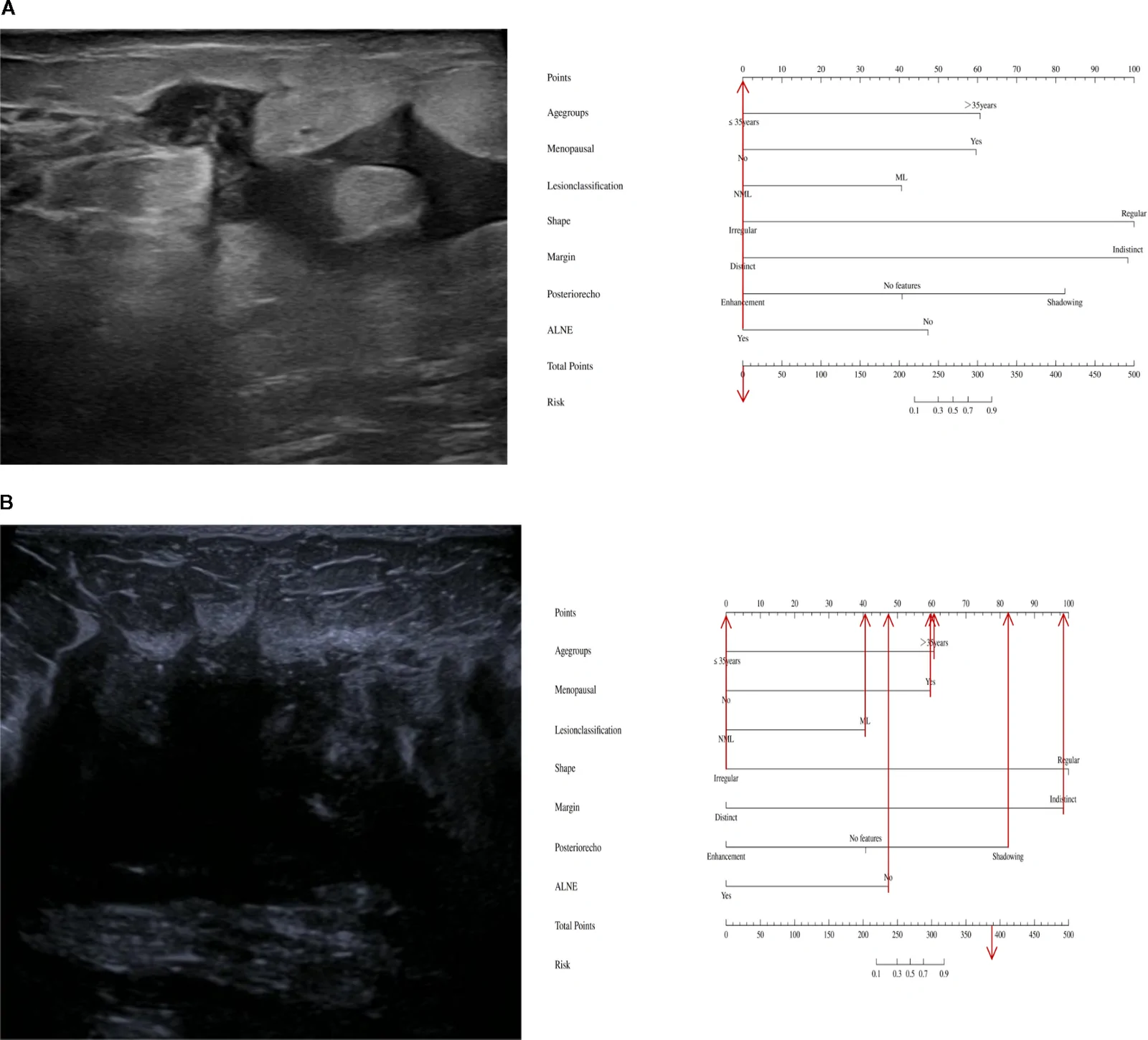
**(a)** Representative case of granulomatous lobular mastitis in a 24-year-old woman (0 points), premenopausal (0 points), with a non-mass lesion on ultrasound (0 points), irregular shape (0 points), circumscribed margin (0 points), posterior acoustic enhancement (0 points), and lymphadenopathy present (0 points). Total score = 0 (0 + 0 + 0 + 0 + 0 + 0 + 0 = 0). Predicted probability of ILC < 0.1. Histopathology confirmed granulomatous lobular mastitis. **(b)** Representative case of invasive lobular carcinoma in a 56-year-old woman (61 points), postmenopausal (59 points), with a mass lesion on ultrasound (41 points), irregular shape (0 points), indistinct margin (98 points), posterior acoustic shadowing (82.5 points), and no lymphadenopathy (47.5 points). Total score = 389 (61 + 59 + 41 + 0 + 98 + 82.5 + 47.5 = 389). Predicted probability of ILC > 0.9. Histopathology confirmed invasive lobular carcinoma.

Variables with a P value <0.05 in the univariate analysis were selected as candidate variables and entered into the multivariable logistic regression analysis. This analysis identified age group, menopausal status, lesion classification, shape, margin characteristics, posterior echo, and ALNEas independent predictors for differentiating GLM from ILC (*P* < 0.05). Detailed results are summarized in **Table 3**. Based on the multivariate regression results, a nomogram was constructed to facilitate differential diagnosis of GLM and ILC (**Figure 2**). The Hosmer-Lemeshow test indicated good calibration for both training (*χ²* = 8.8093, *df* = 8, *P* = 0.3586) and validation (*χ²* = 9.6019, *df* = 8, *P* = 0.2941) sets, with all P values > 0.05.The model incorporated: shape, posterior echo, margin, menopausal status, ALNE, age group, and lesion classification. Feature importance was further evaluated using Shapley Additive Explanations (SHAP), as illustrated in **Figure 3**. SHAP-based risk stratification of conventional ultrasound and clinical features revealed that regular shape, posterior attenuation, ill-defined margins, postmenopausal status, age >35 years, mass-forming lesions, and absence of ALNE were the predominant predictors for ILC. In descending order of SHAP importance, regular shape contributed the most, followed by posterior echo features, margin characteristics, menopausal status, ALNE, age group, and lesion classification.Based on this nomogram, GLM and ILC can be differentiated based on combined clinical and ultrasonographic indicators (**Figure 4**: 4a, GLM example; 4b, ILC example).

**Table 3:** Multivariate logistic regression analysis.

Variables	B	SE	Z		OR (95% CI)
Age groups					
≤ 35 years					1.00 (Reference)
> 35 years	2.70	0.73	3.70	**< 0.001**	14.83 (3.56~61.81)
Menopause					
No					1.00 (Reference)
Yes	2.65	0.65	4.05	**< 0.001**	14.16 (3.92~51.09)
Lesion classification					
NML					1.00 (Reference)
ML	1.80	0.60	3.00	**0.003**	6.07 (1.87~19.74)
Shape					
Regular					1.00 (Reference)
Irregular	–4.44	0.94	–4.74	**< 0.001**	0.01 (0.00~0.07)
Margin					
Distinct					1.00 (Reference)
Indistinct	4.37	0.90	4.84	**< 0.001**	79.38 (13.49~467.18)
Posterior echo					
No features					1.00 (Reference)
Enhancement	–1.81	0.75	–2.42	**0.016**	0.16 (0.04~0.71)
Shadowing	1.85	0.57	3.23	**0.001**	6.36 (2.07~19.50)
ALNE					
No					1.00 (Reference)
Yes	–2.10	0.52	–4.07	**< 0.001**	0.12 (0.04~0.34)

*Bold：P<0.05*

### 3.4 Nomogram evaluation

**Figure 5:**
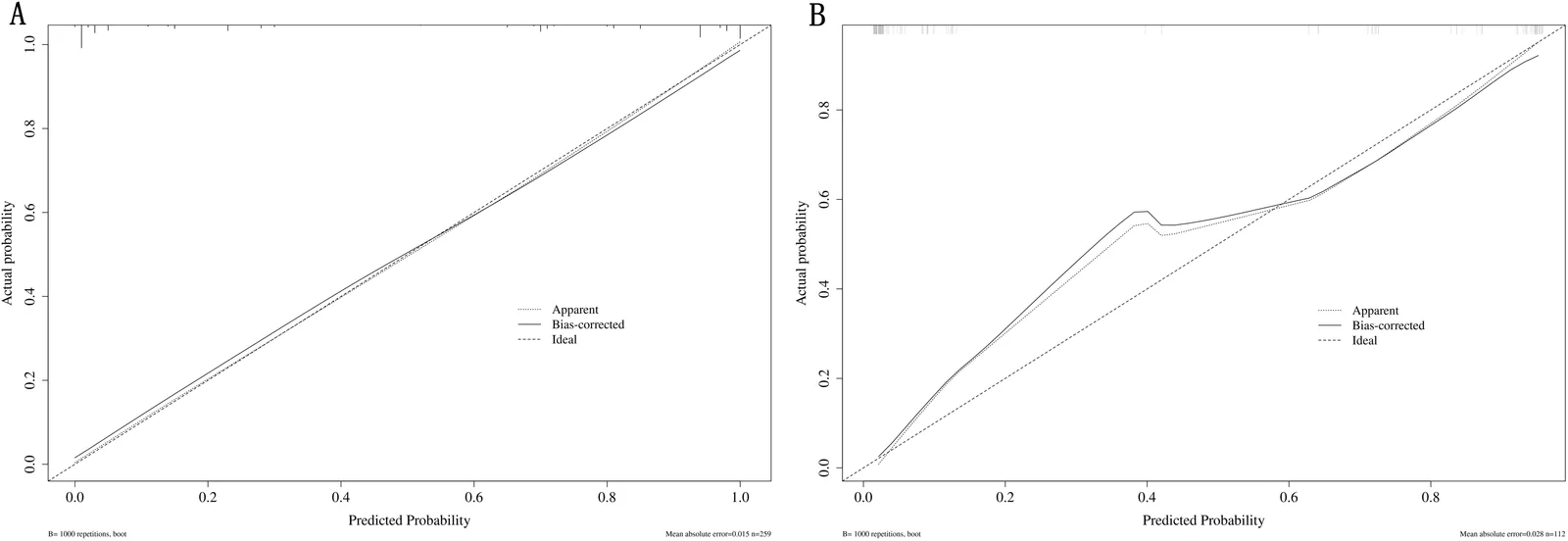
ROC curves for the nomogram in **(a)** training and **(b)** validation sets.

**Figure 6:**
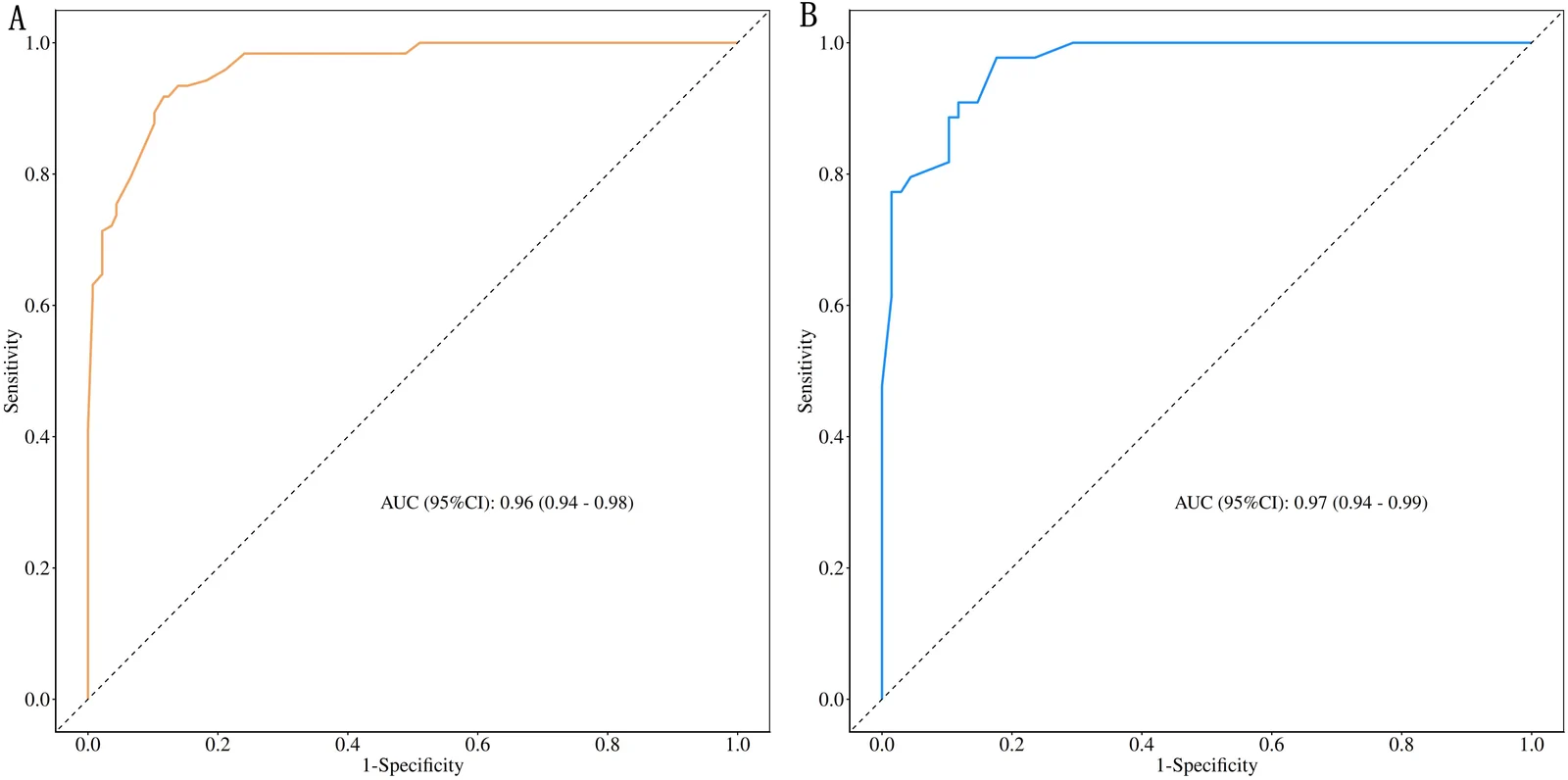
Calibration curves for the nomogram in **(a)** training and **(b)** validation sets.

**Figure 7:**
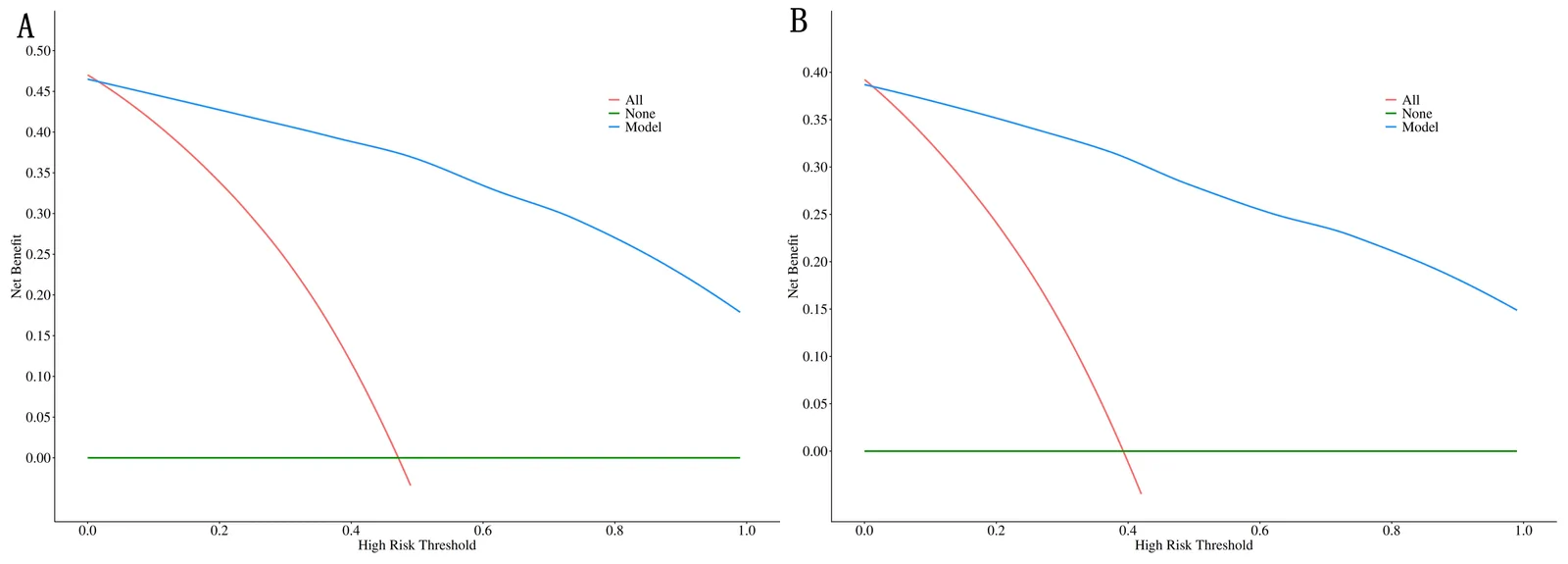
DCA for the nomogram in **(a)** training and **(b)** validation sets.

ROC curve analysis indicated strong diagnostic performance of the nomogram, with an AUC of 0.96 (95% CI: 0.94–0.98) in the training cohort and 0.97 (95% CI: 0.94–0.99) in the validation cohort (**Figure 5**). Calibration curves indicated good agreement between the predicted probabilities and observed outcomes in both the training and validation sets (**Figure 6**). The calibration intercept was −0.0014 (ideal value, 0), the calibration slope was 1.0025 (ideal value, 1), and the coefficient of determination (R²) was 0.9893, confirming a highly consistent linear relationship between the predicted probabilities and the actual event rates. The Hosmer–Lemeshow test (*χ²* = 8.8093, *df* = 8, *P* = 0.3586) and a Brier score of 0.075 further supported the good calibration of the model, reflecting low prediction error and demonstrating reliable clinical calibration. DCA indicated that the nomogram provided a superior net clinical benefit across a clinically relevant range of threshold probabilities in both cohorts (**Figure 7**). DCA results show that both Training and Validation Sets provide significantly higher net benefits than the “biopsy all” or “biopsy none” strategies across the 0.2–0.4 threshold range, confirming their robust clinical decision support utility in this key interval. **Table 4** summarizes the predictive performance of the nomogram model across different cohorts.

**Table 4:** Comparison of diagnostic performance of the nomogram model in the training set and validation set.

Cohort	AUC (95%CI)	Accuracy (95%CI)	Sensitivity (95%CI)	Specificity (95%CI)	PPV (95%CI)	NPV (95%CI)	Cut off
Training set	0.96 (0.94-0.98)	0.90 (0.86-0.93)	0.92 (0.87 - 0.97)	0.88 (0.83 - 0.94)	0.88 (0.82 - 0.93)	0.92 (0.88 - 0.97)	0.579
Validation set	0.97 (0.94-0.99)	0.89 (0.82-0.94)	0.89 (0.79 - 0.98)	0.90 (0.82 - 0.97)	0.85 (0.74 - 0.95)	0.92 (0.86 - 0.99)	0.579

## 4 Discussion

GLM and ILC, both lesions originate from the lobular units of the breast; however, they represent fundamentally distinct pathological entities. GLM is a benign inflammatory disorder, and ILC is a malignant neoplasm. Their biological behavior, therapeutic strategies, and prognostic outcomes differ markedly (11). The incidence of GLM has demonstrated an increasing trend in recent years and is frequently associated with a prolonged and recurrent clinical course (12). The considerable overlap in clinical manifestations and imaging findings between GLM and ILC presents a significant diagnostic challenge in the preoperative setting, with definitive diagnosis often relying on histopathological examination. However, the time required for biopsy and pathological confirmation may delay treatment decisions. Therefore, improving the accuracy of preliminary differentiation using ultrasonography is of substantial clinical importance, as it may reduce diagnostic delay and alleviate patient anxiety. In this study, both univariate and multivariate logistic regression analyses were performed to identify independent predictors; a nomogram was subsequently developed based on the relative contribution of these variables. The final model incorporated age group, menopausal status, lesion classification, shape, margin characteristics, posterior echo, and ALNE, reflecting differences in host characteristics, lesion morphology, and biological behavior between GLM and ILC. With the EPV value of 17.4, the risk of overfitting was low. Bootstrap internal validation revealed only a minimal decrease in the C-statistic, further confirming the model’s robustness.

In the present study, irregular shape (odds ratio [OR] = 0.01), posterior acoustic enhancement (OR = 0.16), and ALNE (OR = 0.12) were identified as predictive features promoting GLM. Although an irregular shape was observed in both groups, it was significantly more prevalent in GLM (91.97% versus 77.87%) and remained statistically significant in univariate and multivariate analyses. This finding may be attributable to GLM pathogenesis and the associated local granulomatous reaction (13, 14). Granulomatous inflammation induces a surrounding inflammatory response, characterized by inflammatory cell infiltration, microabscess formation, and varying degrees of fibrosis, which collectively disrupt the normal breast architecture and result in more irregular lesion contours.

Posterior acoustic enhancement was more indicative of GLM, whereas posterior acoustic shadowing was more commonly associated with ILC, serving as an important discriminative feature. These differences are closely related to the histopathological composition of the lesions (15). GLM lesions often contain fluid components and loosely arranged connective tissue, leading to reduced ultrasound attenuation and increased sound transmission, manifesting as posterior enhancement. In contrast, ILC is characterized by diffuse infiltration of tumor cells accompanied by dense desmoplastic reaction and collagen deposition. This creates a high-density, high-scattering medium, and the diffuse infiltration along interlobular stroma without complete architectural destruction leads to strong sound attenuation, resulting in increased acoustic attenuation and posterior shadowing (16). Variability in posterior acoustic patterns observed in some cases may be related to differences in the degree of fibrosis or liquefaction within the lesion. Patients with GLM were more likely to present with ALNE. ALNE was more frequently observed in patients with GLM, which is closely associated with its inflammatory nature. In GLM, inflammatory processes originating in the lobular units may extend through lymphatic channels to the axillary nodes, leading to hyperplasia and enlargement. In contrast, lymph node involvement in ILC is primarily due to metastatic spread via lymphatic drainage (17, 18). Moreover, the sonographic characteristics of enlarged lymph nodes differ between the two conditions (19). In GLM, lymph nodes typically exhibit preserved fatty hilum and uniform cortical thickening, whereas in ILC, lymph nodes may demonstrate cortical asymmetry, round morphology, and sometimes loss of the normal corticomedullary differentiation.

In contrast, age > 35 years (OR = 14.83), postmenopausal status (OR = 14.16), indistinct lesion margins (OR = 79.38), and presentation as a mass lesion (OR = 6.07) were strong predictors for ILC. The mean age of GLM onset in this study was 34.40 ± 6.61 years, compared to 50.29 years for ILC (*P* < 0.01). Age group and menopausal status also displayed significant differences between groups (*P* < 0.01), consistent with previous reports indicating that ILC tends to occur in older, predominantly postmenopausal women, whereas GLM predominantly affects women of reproductive age (5, 12, 20). The inclusion of age and menopausal status in the nomogram enhances predictive capability.

Indistinct margins, encompassing spiculation, angular margins, and indistinctness, demonstrated the highest predictive value for ILC (OR = 79.38). This feature is closely associated with the infiltrative growth pattern of ILC. Loss of E-cadherin expression allows tumor cells to infiltrate diffusely along stromal and adipose tissue without forming a well-defined tumor boundary. This infiltration, often accompanied by a desmoplastic stromal reaction, produces the classic sonographic signs of spiculation, angular margins, and indistinct borders (6). In contrast, although GLM may present with irregular margins due to inflammation and edema, it rarely produces true spiculated margins, as fibrosis is generally less pronounced and more localized (14).

The proportion of mass-type lesions differed significantly between GLM and ILC in both univariate and multivariate analyses (OR = 6.07), demonstrating discriminative value. These distinct imaging presentations stem from underlying pathological differences (6, 21, 22). In ILC, diffuse tumor infiltration combined with stromal reaction leads to the formation of a structurally identifiable, albeit often ill-defined, hypoechoic mass (23). In contrast, GLM is characterized by inflammatory infiltration centered on the lobules with microabscess formation and coalescence, often manifesting on ultrasound as non-mass-like abnormalities, including hypoechoic areas, architectural distortion, dilated ducts, or sinus tracts, with margins that may fluctuate with disease activity (24, 25). Essentially, a mass lesion reflects the structural space-occupying nature of ILC’s tumor proliferation and stromal response, whereas a non-mass lesion corresponds to the primarily infiltrative inflammatory pathology of GLM. Consequently, in the nomogram, presentation as a mass lesion is a factor favoring ILC diagnosis.

Interestingly, traditional sonographic indicators of malignancy, including aspect ratio, calcification, and hyperechoic halo, were not retained in the final model. This may be attributed to overlapping features between the two conditions. First, regarding aspect ratio, the “non-parallel” orientation typically associated with malignancy may be observed in both conditions. While ILC grows infiltratively along tissue planes, GLM-related inflammation and edema can also restrict vertical growth, resulting in a similar lack of significant difference in aspect ratio distribution (*P* > 0.05) (26, 27). Second, although calcifications, particularly microcalcifications, are an important feature of ILC (28), GLM can also present with coarse or scattered calcifications secondary to necrosis and inflammation. From a histopathological perspective, ILC cells are dyscohesive due to E-cadherin loss and form few glandular lumina, leading to a lower incidence of microcalcifications than in invasive ductal carcinoma, when microcalcifications do occur, they are often subtle and amorphous. Despite calcification displaying a statistically significant difference in univariate analysis (*P* < 0.001), its diagnostic contribution within the multivariate model was insufficient for inclusion, warranting further investigation. Third, the hyperechoic halo, which reflects peritumoral edema or desmoplasia in malignancy (29), can appear as a thick or irregular echogenic rim around GLM lesions due to inflammatory infiltration and fibrosis. Conversely, due to the single-file infiltration pattern of ILC, the halo may be incomplete or absent. The limited discriminatory power of this feature in our cohort likely precluded its inclusion in the final model.

This study has several limitations. First, it was a retrospective analysis with a complete-case dataset with no missing values, and selection bias may not be entirely excluded. Second, this was a single-center retrospective study, and the model underwent only internal validation, without external validation, which may limit the generalizability of our findings. Future multicenter external validation will be pursued to further substantiate our findings. Third, to enhance clinical applicability and general accessibility, the study incorporated only clinical and conventional B-mode ultrasound features. Advanced techniques such as contrast-enhanced ultrasound (CEUS) and elastography were not included, which may have limited the model’s ability to capture information regarding lesional hemodynamics and tissue stiffness, potentially capping further improvements in diagnostic performance.

In conclusion, the nomogram model constructed in this study integrates seven independent predictors: age group, menopausal status, lesion classification, shape, posterior echo, margin characteristics, and axillary lymph nodes enlarged. Although this model requires external validation, it still offers an effective, non-invasive, quantitative tool for differentiating GLM from ILC and determine the need for biopsy, while also providing a valuable reference for guiding individualized therapeutic strategies in clinical practice.

## Data Availability

The original contributions presented in the study are included in the article/Supplementary Material. Further inquiries can be directed to the corresponding author.
